# Understanding patterns of internal migration during the COVID‐19 pandemic in Spain

**DOI:** 10.1002/psp.2578

**Published:** 2022-06-16

**Authors:** Miguel González‐Leonardo, Antonio López‐Gay, Niall Newsham, Joaquín Recaño, Francisco Rowe

**Affiliations:** ^1^ International Institute for Applied Systems Analysis, Wittgenstein Centre for Demography and Global Human Capital Vienna Austria; ^2^ Department of Geography Universitat Autònoma de Barcelona Barcelona Spain; ^3^ Centre d'Estudis Demogràfics Barcelona Spain; ^4^ Department of Geography and Planning University of Liverpool Liverpool UK

**Keywords:** COVID‐19, internal migration, rural areas, Spain, urban exodus, urban hierarchy

## Abstract

Existing empirical work has analysed the impacts of COVID‐19 on mortality, fertility and international migration. Less is known about the ways in which the COVID‐19 pandemic has influenced the patterns of internal migration. Anecdotal reports of mass migration from large cities to less populated areas have emerged, but lack of data has prevented empirically assessing this hypothesis. Drawing on geographically granular administrative population register data, we aim to analyse the extent of change in the patterns of internal migration across the urban hierarchy in Spain during 2020. Our results show a decline of 2.5% in the number of internal migration moves, particularly during the early stages of the pandemic, returning to pre‐pandemic levels in late 2020. Results also reveal unusually large net migration losses in core cities and net migration gains in rural areas. Net migration losses in cities and gains in rural areas particularly accumulated following the elimination of the strict lockdown measures in June. Yet, these net losses and gains trended to pre‐pandemic levels in late 2020, and movements between cities, and between cities and suburbs, continued to dominate the internal migration system. Thus, while the COVID‐19 pandemic exerted notable changes in the geographic balance of internal migration flows, these changes appear to have been temporary and did not significantly alter the existing structures of the national migration system.

## 1 INTRODUCTION

As fertility and mortality stabilise, migration has become the main driver of population change shaping patterns of human settlement both between and within countries (Bell et al., 2015). Together with international migration, internal migration is the primary agent redistributing population between subnational boundaries (Bell et al., 2015). Internal migration underpins the efficient functioning of the economy by bringing knowledge and skills to the locations where they are needed and is essential to social well‐being by enabling individuals to pursue their goals and aspirations (Van Ham et al., 2001). Internal migration is widely regarded as an integral component of human development (Klugman, 2009; Skeldon, 1997).

In the wake of the COVID‐19 pandemic, government stringency measures globally resulted in a major disruptive shock to the human mobility system of countries, constraining international travel (Guadagno, 2020; IOM, 2020) and local daily mobility patterns (Duque et al., 2021). The extent to which these interventions have impacted internal migration movements is less well understood. Reflecting on the postpandemic future of big cities, hypotheses have emerged on the ways in which COVID‐19 may have impacted internal migration patterns.

During early phases of the pandemic, reports of an ‘urban exodus’ emerged with rampant speculation that this trend would persist post‐COVID‐19 (e.g., Marsh, 2020; Nathan & Overman, 2020). As COVID spread across the world and we started learning about its severity, mounting fear and anxiety resulted in a need to minimise social contact and avoid crowded spaces. Globally, big cities became the first epicentres of infection (Pomeroy & Chainey, 2020). High population density, strong air connectivity and spatial concentration of public‐facing jobs serve as conducive mechanisms for the clustering of COVID‐19 cases in large cities (Bhadra et al., 2021; Florida et al., 2021). These factors created dense networks of social interaction facilitating community transmission and geographical spread of COVID‐19 (Brandén et al., 2020).

Emerging anecdotal evidence indicates that big city residents moved to second homes, vacation towns, suburban areas and other smaller cities (e.g., Hughes, 2020; Marsh, 2020; Paybarah et al., 2020). The pandemic has brought about changes that have allowed nonwork related considerations to become more prominent in the residential decision making process (Hernández‐Morales et al., 2020; Nathan & Overman, 2020). In addition to the existing urban pressures from air pollution, housing affordability and crime, big city residents were confined to small living spaces, with limited capacity to accommodate remote work, home‐schooling and other daily activities during lockdowns. Business shutdowns and social distancing measures stripped away the effervescence of social interaction, vibrant urban spaces and many were pushed into unemployment (Blustein et al., 2020; Honey‐Rosés et al., 2020). Additionally, telework reduced the need for frequent commuting, and hence, living in close proximity to work. These changes are believed to have reduced attachments to big cities in preference for larger, cheaper and less crowded residential areas during the pandemic, or seek for a refuge in family homes (Florida et al., 2021).

While these changes are expected to alter micro‐level household decision choices, they are not expected to significantly reshape the existing macro‐level patterns of national population settlement and economic systems (Florida et al., 2021). Big cities have successfully weathered previous pandemics (Glaeser, 2020) and are likely to remain attractive places to live. Agglomeration economies are a fundamental feature of cities. Cities facilitate the clustering of talent and economic assets, consumer base, face‐to‐face interaction and diversity that are key to fostering innovation, creativity and economic growth (Storper & Venables, 2004). New forms of hybrid work are a likely permanent outcome of the pandemic (Alexander et al., 2021). Telework is a poor substitute for high‐contact, knowledge‐based work. At the same time, more remote locations may not have the digital infrastructure and diversity of services required to cater for city residents—and initiatives are already underway to make cities more resilient to future pandemics (Eltarabily & Elghezanwy, 2020; Moreno et al., 2021; Sharifi & Khavarian‐Garmsir, 2020).

Thus, while anecdotal evidence has emerged about large out‐migration flows from big cities, little is known about the extent and durability of these patterns. The lack of suitable data has prevented the analysis of internal migration movements across the rural‐urban continuum at a national scale. We had exclusive access to controlled micro‐data covering all reported changes in residential address in Spain between January 2016 and December 2020. Drawing on these data, we analysed the extent and persistence of changes in internal migration patterns in Spain during 2020. Spain was one of the first hardest hit countries by the COVID‐19 pandemic in 2020, with one of the highest excess mortality records across Europe (Eurostat, 2022), and a strict lockdown from mid‐March to late April. We specifically seek to address the following set of questions:

1. To what extent did people leave cities, and moved to less populated areas?
2. How did these patterns vary across cities during the pandemic?
3. What have been the main destinations for migration flows from cities?
4. How have the patterns of internal migration changed over the course of the pandemic? Have the observed changes been short‐term? Did they persist over 2020?

The rest of the paper is structured as follows: Section 2 discusses anecdotal evidence indicating how COVID‐19 may have impacted internal migration and the contemporary patterns of internal migration before the pandemic across the Spanish urban hierarchy. Section 3 described the data and method used for the analysis. Section 4 presents the results before discussing the key findings and offering some concluding remarks in Section 5.

## 2 BACKGROUND

### 2.1 Emerging evidence on internal migration patterns during COVID‐19

As COVID‐19 expanded throughout the world in February and March of 2020, anecdotal evidence of an ‘urban exodus’ from big cities emerged in many western societies (Davies, 2021; Marsh, 2020; Matheson et al., 2020). In early stages of the pandemic, little was known about the virus, and globally connected cities were hit hardest (OECD, 2020; The Economist, 2022). By November 2020, approximately 95% of all the reported infections and fatalities had occurred in cities (Pomeroy & Chainey, 2020). Rampant speculation was linked to newspapers’ headlines claiming ‘the end of cities’ (e.g., Cavendish, 2020; Kimmelman, 2020). In the United Kingdom, the number of online inquiries from residents in the 10 largest cities looking for a village property was reported to increase by 126% in June–July 2020 relative to the same period in 2019 based on data from the property website Rightmove (Marsh, 2020). Increases in real estate transactions outside cities were also linked to city residents moving to smaller towns or villages in France (Sagnard, 2021). In the United States, a rise of 30 percentage points in the number of households moving from large metropolitan areas was reported based on data from mail‐forwarding requests and credit report data (Hughes, 2020; Whitaker, 2021). Australian Bureau of Statistics data indicate a net loss of 11k people from Australian capital cities in the September quarter of 2020 (Davies, 2021). Recent empirical evidence also saw an increase of net‐migration losses in some cities of Japan, Germany, Sweden and the United Kingdom (Fielding & Ishikawa, 2021; Rowe et al. 2022; Stawarz et al., 2022; Vogiazides & Kawalerowicz, 2022).

Various factors contributed to the ‘urban exodus’ narrative with COVID‐19 exposing key shortcomings of living in big cities. Facilitated by high air‐travel connectivity, job density and spatial concentration of public‐facing jobs, big cities became early global epicentres of COVID‐19 infections during the early phases of the pandemic (Florida et al., 2021; Rodríguez‐Pose & Burlina, 2021). Prepandemic housing affordability and poor housing conditions have been consistent urban challenges in big cities. Coupled to these challenges, lockdowns, social distancing, remote work and homeschooling have reportedly added greater pressure for families living in small and crowded living spaces, to move out of cities in the look for more space and affordable housing (Hughes, 2020; Marsh, 2020). Teleworking and increased familiarity, and use of online shopping, reduced the need for commuting and living in proximity to work locations and shops. Business closures removed the effervescence of urban entertainment, leisure and social spaces, and triggered a rapid spike in unemployment in many countries during 2020 as nonessential, public‐facing work shut down (King, 2020; Smith et al., 2021).

COVID‐19 is claimed to have fuelled migration from big cities to rural areas, suburbs and smaller cities (e.g., Hughes, 2020; Sagnard, 2021). Technologies to facilitate remote work, such as video conferencing, shared documents, instant messaging and cloud computing are now widely available. Virtual services, like video streaming and social media platforms offer access to some of the cultural effervescence and community that has drawn people to big cities. Online shopping platforms, such as Amazon and Ebay provide an opportunity to buy and ship products from distant locations.

However, internal migration movements during the pandemic seem to have been over relatively short distances. Preliminary evidence from the United States and Sweden indicates that the vast majority of movements from big cities during the pandemic has been towards the suburbs of those very cities, as opposed to smaller, remote cities and towns (e.g., Hughes, 2020; Ramani & Bloom, 2021; Vogiazides & Kawalerowicz, 2022), though urban residents have also moved to neighbouring areas, second residences, holiday destinations and other cities (Quealy, 2020). In Australia, larger cities have been the primary destination of migration from cities of similar size (Davies, 2021). In both countries COVID‐19, does not seem to have fundamentally altered the pre‐existing national structure of the net internal migration balances. However, it seems to have accelerated relocation decisions that were already in motion prepandemic (Davies, 2021; Kolko et al., 2021).

Persuasive cases have been made against headlines speculating about the end of cities. Past pandemics wreaked havoc and substantially influenced medical, cultural, political and urban design changes, but they have not dented the key role that cities play in society (Glaeser, 2020). For instance, the Black Plagues of the 14th century killed one‐third of the population in Europe and the Middle East (Dols, 2019; Pamuk, 2007). The Cholera outbreaks of the 19th century decimated major cities across the world, including, London, Paris, Moscow, Hamburg, New York and Madrid (Evans, 1988). Yet, large cities have continued to be important gravitational forces of population concentration.

Cities are critical engines of innovation, economic growth and prosperity. They enable the emergence of agglomeration economies. Concentration in cities facilitates the exchange of goods, knowledge, information and ideas by reducing transportation and communication costs, offering abundant critical mass, and fostering strong firm linkages (Glaeser, 2010). A fundamental ingredient underlying these benefits is the face‐to‐face interaction that urban concentrations enable (Storper & Venables, 2004). While routine, codified activities can be more easily communicated and performed virtually from remote locations, complex, innovative and less familiar tacit knowledge, tasks and ideas are facilitated by face‐to‐face contact (Storper & Venables, 2004). This is a fundamental reason why the advent of internet communication has not led to the geographic dispersal of urban agglomerations and ‘the death of distance’, despite enabling various forms of complex communication to occur at a distance (Tranos & Nijkamp, 2013).

At the same time, rural and remote areas may lack the infrastructure and services needed. They do not offer the vibrancy and sophistication of entertainment, cultural and convenient services that urbanites are used to. Additionally, telework is likely to be a permanent outcome of the COVID‐19 pandemic. Yet, poor broadband connectivity in rural and remote locations has remained a key challenge across most countries in the world (Chen & Wellman, 2004). Not all forms of work can be done remotely, including high‐touch, public facing work providing essential (e.g., healthcare and education services) and nonessential (e.g., restaurants, bars and clothing stores) services; essential, nonpublic facing work related to construction, infrastructure and maintenance; and knowledge‐intensive activities requiring high‐level abstraction and cognitive capacity, such as teaching and networking (Florida et al., 2021). Online work fatigue has become a new phenomenon, with the widespread use of ‘Zoom fatigue’ (Wiederhold, 2020). Rather than full‐time remote work, hybrid forms of work are more likely to stay post‐pandemic, requiring flexibility to combine office and online presence (Haag, 2021). This may entail a need for reliable broadband connectivity and accessibility to employment centres.

Thus, speculations during early phases of the pandemic pointed to an ‘urban exodus’ as COVID‐19 cases and deaths surged in large cities. Emerging evidence suggests that the effects from the pandemic have reverberated through the internal migration system of countries across the world prompting residential relocations from cities (Fielding & Ishikawa, 2021; Stawarz et al., 2022; Vogiazides & Kawalerowicz, 2022). Yet, these reverberations are not expected to have significantly redrawn the internal migration system (Brown & Tousey, 2021). Rather, they may have accelerated existing migration trends, and cities are expected to bounce back and remain major centres of agglomeration postpandemic. Thus far, however, existing evidence remains scarce. We therefore seek to offer empirical evidence assessing the ways in which the Spanish internal migration system has weathered during the pandemic in 2020.

### 2.2 Contemporary trends of internal migration across the Spanish urban hierarchy

Spain stands out as one of the least mobile countries in Europe (Bell et al., 2015; Bernard & Vidal, 2020; Rowe et al., 2019). According to *Estadística de Variaciones Residenciales* (EVR) data, an overall rate of internal migration of 3.3% indicates that only around 3% of the Spanish population changed municipality of residence during the 2016–2020 period. This is similar to the low migration intensities observed in other countries in Southern and Eastern Europe, including Italy, Greece, Poland, Romania, Russia and Czech Republic (Alvarez et al., 2021; Rowe et al., 2020). But, it contrasts with the high levels found in Nordic and Western European countries, with internal migration rates of over 18% in Iceland and France (Rees et al., 2017). Spain also displays one of the lowest levels of internal migration efficiency; that is, the extent to which the national population is redistributed through internal migration. Spain reports an internal migration efficiency of 6.3%, which is below the European mean of 11.4% (Rowe et al., 2020). Such low levels of migration efficiency in the Spanish internal migration system reflects very low levels of population redistribution resulting from migration inflows being closely balanced by outflows.

Precoronavirus low levels of internal migration efficiency and intensity in Spain resulted in a migration system characterised by a pattern of spatial equilibrium (Rowe et al., 2019; Rowe & Patias, 2020). This is a system in which migration flows across the national urban settlement are closely balanced, resulting in limited population redistribution (López‐Gay, 2017; Recaño, 2020). Even so, population redistribution occurs with three distinct processes: urbanisation, suburbanisation and counterurbanisation (Gil‐Alonso et al., 2021; González‐Leonardo, 2021; López‐Gay et al., 2020; Torrado et al., 2020). Movements from major core cities to suburbs and population exchanges between cities are a prevalent form of mobility in the Spanish migration system (Bayona & Pujadas, 2020; González‐Leonardo et al., 2019; Rowe & Patias, 2020). Pre‐coronavirus, the migration system in Spain was also characterised by population losses in inland rural areas due to internal migration towards cities (Collantes & Pinilla, 2011; Recaño, 2020). In this paper, we analyse how COVID‐19 has impacted these pre‐existing patterns of internal migration across the urban hierarchy.

## 3 DATA AND METHOD

We used register population data to capture internal migration measuring changes in the municipality of residence. We analysed internal migration during 2020 and used averaged indicators across the 2016–2019 period as a benchmark to determine deviations from pre‐pandemic trends. We analysed the data in four phases. First, we explored out‐, in‐ and net‐migration flows across the urban hierarchy to explore the extent of changes in internal migration from cities to less populated areas. Second, we examined out‐, in‐ and net‐ migration outcomes for five core cities which experienced large population losses during 2020 (Madrid, Barcelona, Valencia, Zaragoza and Bilbao) to identify their key destinations along the urban hierarchy and extent of variations across cities in 2020. Third, we analysed the spatial patterns of internal migration at the municipal geographical scale to identify key changes across the internal migration system and the extent of these variations in less populated areas. Finally, we explored monthly changes in the levels of internal migration flows during 2020 to determine the durability of the internal migration patterns observed during the COVID‐19 pandemic.

### 3.1 Data

We had access to a unique and large administrative data set recording all individual reported changes in place of residence in Spain. We drew on data from official microdata collected by the (EVR) of the Instituto Nacional de Estadística (INE) from 2016 to 2020 for 8131 municipalities. An open access version of this data set is available to researchers, but it does not include information for municipalities with less than 10,000 inhabitants, which equate to 90.7% of all Spanish municipalities. The data set we used includes data for all municipalities. During the lockdown, mainly in the early stages, EVR registrations were affected by underreporting, but the effect of this influence is unclear. On the one hand, closure of some in‐person EVR registration offices may have resulted in under‐reporting, with EVR capturing a lower number of changes in residential locations than those occurring. On the other hand, telematic services were rapidly deployed across the country, enabling digital registration. Additionally, selective local and regional mobility restrictions on nonessential activities were implemented during the last 4 months of 2020 where COVID‐19 cases increased, mostly in cities (La Moncloa, 2021). It may have led to over‐reporting in rural areas caused by potentially untruthful residential registrations to avoid mobility restrictions in cities. Estimating the balance between these registration challenges is not possible as we have no data on the levels of under‐ and over‐reporting. While we do not think these issues invalidate our conclusions, they make the identification of the causes of the observed changes in residence more challenging. It is difficult to determine if EVR registrations indicate a real or a convenient change of residence. Yet, we believe that the data still provides a relatively accurate representation of the internal migration trends during 2020.

### 3.2 Method

As we explained below, our analysis consisted of four stages:

*Stage 1*. To analyse the extent of internal migration from cities, we measured variations in migration intensity and impact across the urban hierarchy. To this end, we classified municipalities into core cities, suburbs, towns and rural areas. Core cities and suburbs are classified based on the official Spanish urban area classification—the *Átlas Estadístico de las Áreas Urbanas* of the Spanish *Ministerio de Transportes, Movilidad y Agenda Urbana* (MITMA). This source only includes urban areas, therefore core cities and their suburbs are listed, but not towns and rural areas. It used several variables to classify municipalities as urban areas, such as administrative criteria, population density, size, trends and structure, urban morphology, housing market, transport infrastructures or labour market characteristics. Core cities represent central municipalities of urban areas and are classified based on administrative criteria, including most Spanish capital cities. We classified municipalities which are not considered as urban areas into towns or rural areas if they are larger or smaller than 10,000 inhabitants, respectively. Table 1 in the Supporting Information Material shows the distribution of municipalities across these areas. In this stage, we used system‐wide indicators to quantify changes in mobility patterns across the national migration system. To capture migration intensity, we estimated the number of people moving in and out of core cities, suburbs, towns and rural areas. To measure the spatial impact of migration, we measured net migration which captures the net balance between in‐ and out‐migration flows.

*Stage 2*. We analysed variations in internal migration patterns across core cities. Focusing on a selection of five core cities (Madrid, Barcelona, Valencia, Zaragoza and Bilbao), we examined changes in out‐, in‐ and net‐migration rates from these cities to their suburbs (according to the MITMA), other urban areas (core cities and suburbs), towns and rural areas. We chose these cities because they represent key population agglomerations in the Spanish settlement system with populations of over 300k, recorded large overall increases in net migration losses during the COVID‐19 pandemic in 2020 (higher than 50% compared to 2016–2019) and provide a diverse overview of changes in local regional internal migration systems. For our analysis, we used city‐specific out‐migration rates (ORs), in‐migration rates (IRs) and net‐migration rates (NRs):

(1) $${\mathrm{OR}}_{i}=\frac{0_{i}}{P_{i}\;}\times 1000,$$

(2) $${\mathrm{IR}}_{i}=\frac{I_{i}}{P_{i}}\times 1000,$$

(3) $${\mathrm{NR}}_{i}=\frac{\left(I_{i}-O_{i}\right)}{P_{i}}\times 1000,$$

where *O_i_* is the number of migrants moving out of the origin area *i*; *I*_*i*_ is the number of migrants moving to the destination area *i*; and *P*_*i*_ is the middle population.

*Stage 3*. We analysed the spatial patterns of internal migration using municipality‐specific ORs, IRs and NRs to identify the location of key internal migration winners and losers during COVID‐19 in 2020 and quantify the demographic impact of out‐migration flows from these cities to less populous areas.

*Stage 4*. We analysed monthly changes in the level of internal migration to assess the continuity of internal migration patterns during COVID‐19 and shed light into future patterns. To establish the extent of changes in internal migration patterns during COVID‐19, we compared monthly internal migration counts in 2020 to those recorded in prepandemic years between 2016 and 2019. This comparison captures monthly seasonal variations in internal migration patterns and enables determining the extent to which observed variations deviate from the long‐term trajectory in internal migration intensity. We also analysed these trends across the urban hierarchy, to assess the extent of variation in the volume of internal migration during COVID‐19 across core cities, suburbs, towns and rural areas.

## 4 RESULTS

### 4.1 Changes in internal migration across the urban hierarchy: Cities as losers and rural areas as winners

**Figure 1:**
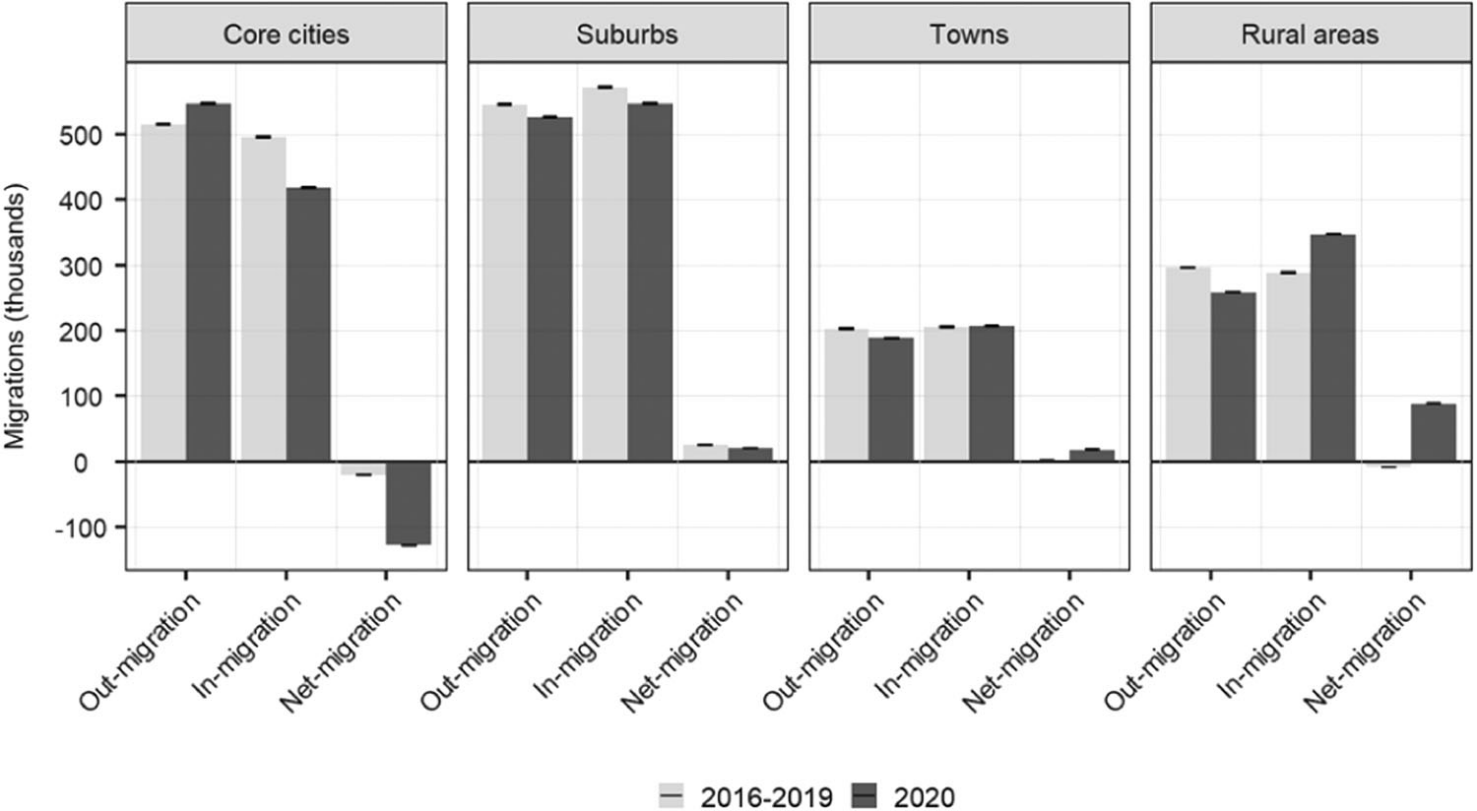
Internal out‐, in‐ and net‐migrations: 2016–2019 (annual average) and 2020. *Note*: The black lines at the top of the bars represent confidence intervals. *Source*: Own elaboration using data from the *Estadística de Variaciones Residenciales* (INE).

Taken together, the number of moves decreased from 1.56 million to 1.52 million (2.5%) between the prepandemic 2016–2019 average and 2020. Although there was not a significant difference in the total number of movements, Figure 1 reveals changes in the patterns of internal migration across the urban hierarchy, particularly in core cities and rural areas. Before the pandemic, a status of spatial equilibrium dominated the national internal migration system, with limited population redistribution as in‐migration flows were closely offset by out‐migration movements.

During the pandemic in 2020, a pattern of population deconcentration emerged reflecting net migration losses in high‐density areas and net migration gains in more sparsely populous locations. Core cities recorded a rise in out‐migration by 6.0% (515k–546k) in 2020, and a drop in in‐migration by 15.4% (494k to 418k). These changes resulted in a population loss due to internal migration of 127k in 2020, representing an increase in annual population loss in core cities. Annual prepandemic net‐migration during the 2016–19 period averaged −20k. By contrast, rural areas recorded a decrease in out‐migration by 12.6% (296k–258k), which was met by a rise in in‐migration by 20.5% (288k–347k), leading to an overall positive net‐migration balance of 88k in 2020. In rural areas, this represents a reversal of prepandemic population losses due to internal migration, and a trend of rising counterurbanisation. Yet, movement between core cities and suburbs continued to dominate the internal migration system in 2020.

### 4.2 Variation on internal migration across cities: Increasing out‐migration from large cities to rural areas

**Figure 2:**
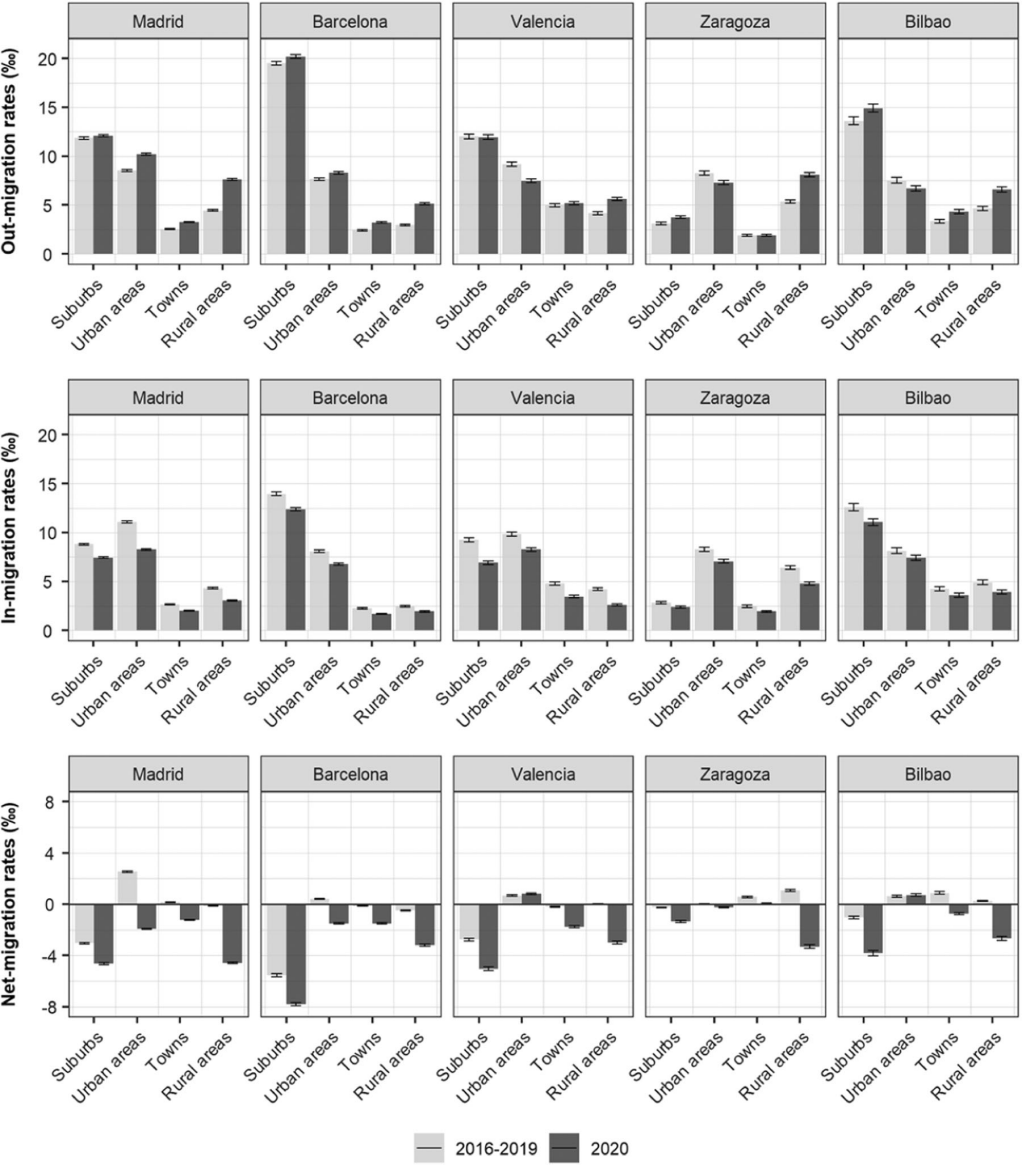
Out‐, in‐ and internal net‐migration rates in selected core cities by type of origin and destination areas (*x*‐axis): 2016–2019 (annual average) and 2020. *Note*: Urban areas include core cities and suburbs within other urban areas; suburbs include municipalities of the own urban area of each core city; confidence intervals are represented at the top of the bars. *Source*: Own elaboration using data from the *Estadística de Variaciones Residenciales* and *Cifras Oficiales de Población* (INE).

We next analyse out‐, in‐ and net‐, migration outcomes for a selection of five core cities. Figure 2 shows out, in‐ and net migration rates in Madrid, Barcelona, Valencia, Zaragoza and Bilbao with their suburbs, other urban areas, towns and rural municipalities. All five core cities display overall negative net migration rates during the pandemic in 2020. These negative net balances exacerbated in the largest core cities, Madrid, Barcelona and Valencia, with rates declining from −0.5%, −5.8% and −2.3% in 2016–19 to −12.4%, −14.0% and −9.0%, respectively (see Table 2 in the Supporting Information Material). In Zaragoza and Bilbao, positive net migration balances in 2016–19 were reversed, declining from 1.4% to −4.9% and 0.7% to −6.5%, respectively. These more pronounced negative net balances were due to a decline of in‐migration and a rise in out‐migration, except for Valencia, where the main cause seems to be an overall decline in in‐migration.

Although rising declines in net migration rates are observed for population exchanges with all types of areas for our sample of cities, the largest drops consistently occurred for migration interactions with rural municipalities, reflecting an acceleration in counter‐urbanisation, especially in Madrid and Barcelona. Figure 2 shows that net migration rates to other urban areas were reversed from positive to negative in the global cities of Madrid and, to a lesser extent in Barcelona, preliminary as a result of declining in‐migration, but also caused by an increase of out‐migration. Yet, suburbanisation is the dominant pattern in the national migration system, except in Zaragoza. Net‐migration with suburbs was negative for all cities in the pre‐pandemic period and further reductions were recorded in 2020 due to a decay in the number of arrivals from suburbs to core cities, particularly in Madrid, Barcelona and Valencia.

### 4.3 Rural areas and coastal towns in close proximity to core cities as key destinations

**Figure 3:**
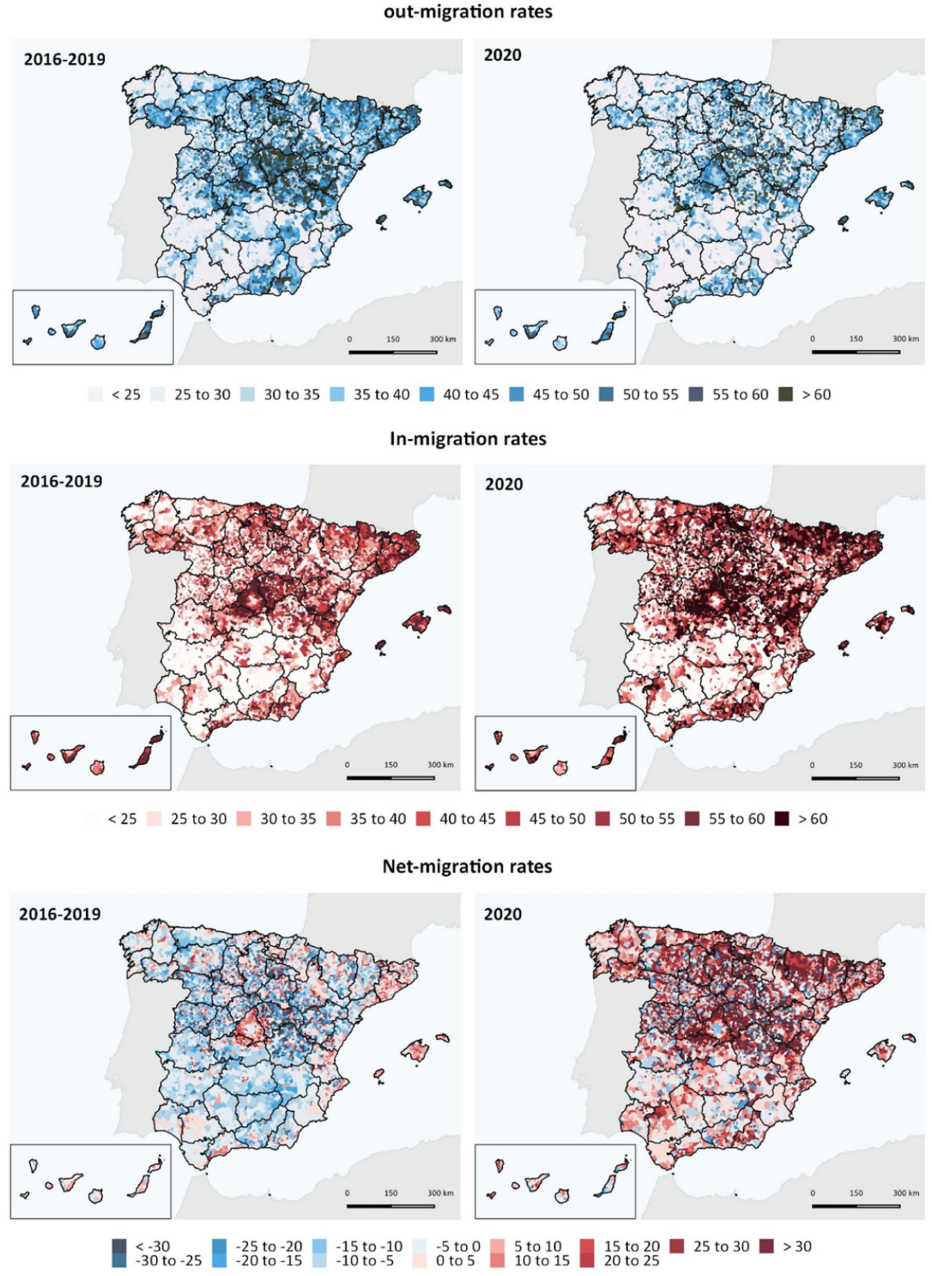
Out‐, in‐migration and net‐migration rates by municipalities (‰): 2016–2019 (annual average) and 2020. *Source*: Own elaboration using data from the *Estadística de Variaciones Residenciales* and *Cifras Oficiales de Población* (INE).

We then explored the spatial patterns of internal migration at the municipal level to understand the general structure of internal migration flows. Figure 3 reports municipality‐specific out‐, in‐ and net‐migration rates for 2020 and the average of the 2016–19 period. Prepandemic, net‐migration rates are predominantly moderate, with net migration gains concentrated in the suburbs of large urban agglomerations, and net migration losses concentrated in rural areas, particularly in east and north of Madrid. During COVID‐19 in 2020, Figure 3 shows the spatial structure of population deconcentration with people away from core cities to suburbs and rural areas. Additionally, it reveals that this pattern of population deconcentration is particularly related to movements from the large urban agglomerations, such as Madrid and Barcelona, to specific locations involving mountain rural areas and certain coastal towns. These areas are known to be popular holiday destinations and concentrate second home residences (Alario et al., 2014; López‐Colás & Módenes, 2004), including areas such as the neighbouring mountain villages of Madrid as a consequence of the arrivals from the capital of Spain (see also Figures 2 and 3 in the Supporting Information Materials); those in the Pirineo Catalán mountain and coastal towns in the north of the Mediterranean, caused by internal migrations from Barcelona; the Pirineo Aragonés mountain due to inflows of residents from the city of Zaragoza; and mountain villages in the north of Burgos where in‐migration from Bilbao increased. In these rural areas, increasing arrivals from cities had a great demographic impact during the pandemic.

### 4.4 Internal migration over the pandemic. Are changes likely to endure after COVID‐19?

**Figure 4:**
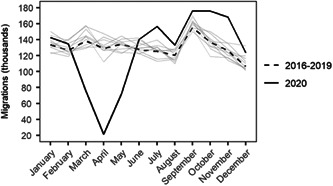
Internal migrations by months in 2016–2019 (annual average) and 2020. *Note*: Grey lines represent years from 2010 to 2019. *Source*: Own elaboration using data from the *Estadística de Variaciones Residenciales* (INE).

As indicated in Section 4.1, the overall number of internal migrants dropped by 2.5% between 2016–19 and 2020. Figure 4 shows that this drop occurred during March–May in 2020, as COVID‐19 spread and a national lockdown was enacted. Following the end of the lockdown in June, the number of internal migrants increased, exceeding the prepandemic levels until November. In December, however, values returned to prepandemic levels.

**Figure 5:**
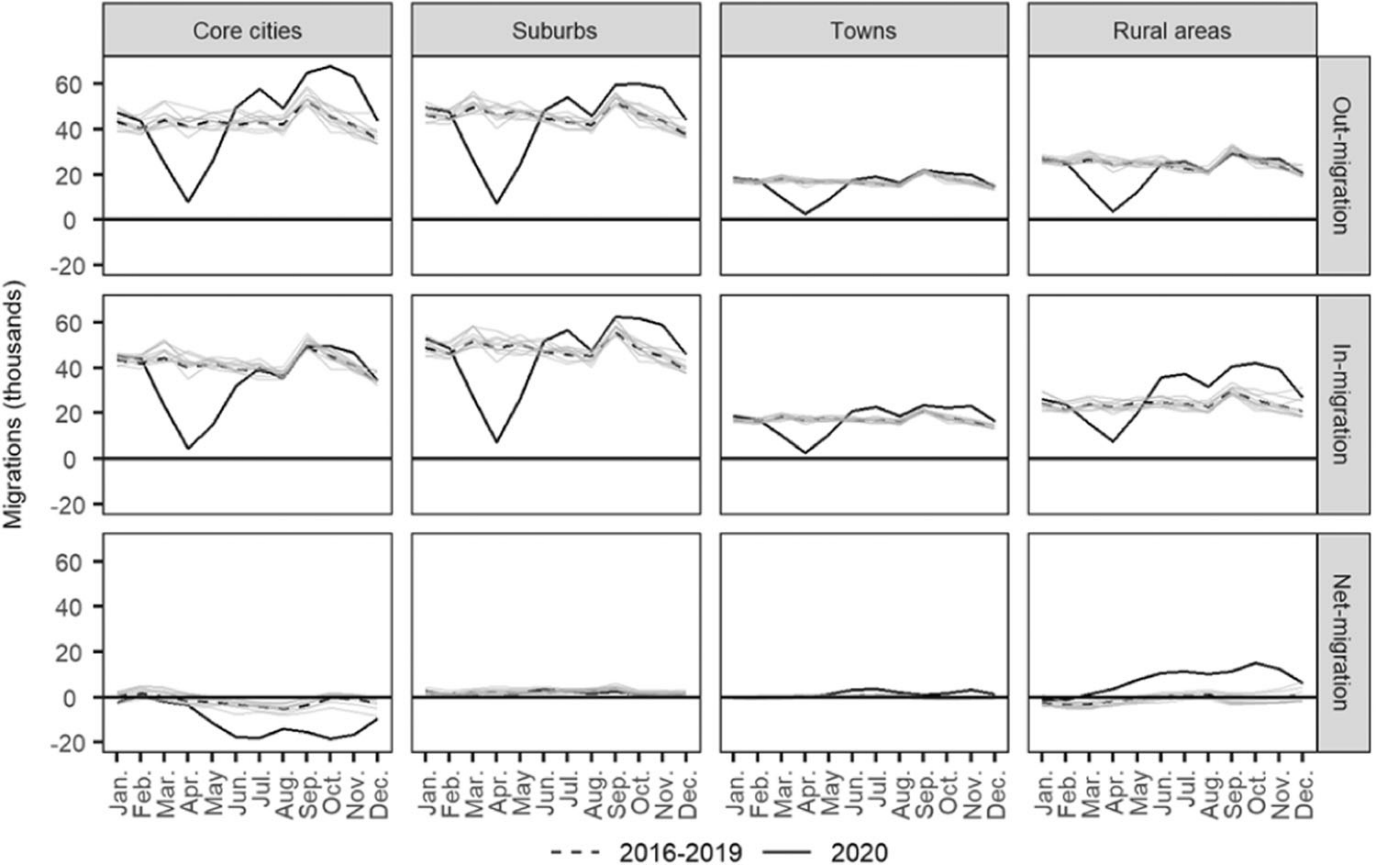
Internal out‐, in‐ and net‐migrations by months and territorial typology: 2016–2019 (annual average) and 2020. *Source*: Own elaboration using data from the *Estadística de Variaciones Residenciales* (INE).

These changes conceal distinctive temporal patterns across core cities, suburbs, towns and rural areas. While all areas recorded a notable drop in in‐migration and out‐migration during March–May, variations in the intensity and recovery of these outcomes during June and November resulted in distinctive net migration balances (Figure 5). Core cities enced a continuing loss of population through internal migration from May to November, driven primarily by out‐migration post April. Relative to prepandemic levels, in‐migration registered little variation with levels similar to those observed for the 2016–2019 period. Net migration balances in suburbs and towns were virtually zero with in‐migration closely balancing out‐migration. In the case of suburbs, this resulted from comparable drops and rises in out‐ and in‐migration levels, while out‐migration and in‐migration levels in towns converged to pre‐pandemic levels following a decline during March and April. As we know, the key gainers of internal migration losses in core cities were rural areas. Figure 5 reveals that net migration gains occurred during May–November as core cities recorded continuing population losses, converging to prepandemic levels in December. Taken together, this evidence suggests that changes in internal migration patterns during COVID‐19 may have been temporary with trends returning to prepandemic levels in late 2020.

## 5 DISCUSSION AND CONCLUSION

During the early phases of the COVID‐19 pandemic, anecdotal evidence of an urban exodus emerged. This hypothesis suggests that the concentration of COVID‐19 in cities, teleworking, lockdowns in poor housing conditions and the shutdown of urban vibrancy led to large migration outflows from cities to less populated areas (Davies, 2021; Marsh, 2020; Matheson et al., 2020). Our results revealed that sudden changes in internal migration patterns occurred across the Spanish urban hierarchy during the COVID‐19 pandemic in 2020. In core cities, out‐migration increased by 6% and in‐migration decreased by 15.4%, resulting in a population loss due to internal migration. Conversely, rural areas recorded a 12.6% decrease in out‐migration and a 20.6% increase in in‐migration, leading to a positive net‐migration balance. Minor changes were observed across suburbs and towns. While these net migration outcomes have a modest impact on the population structure of cities, they resulted in significant expansions in the local population of sparsely populated rural areas. Despite these changes, most migration movements continued to occur between core cities and suburbs, or between different urban areas. These results indicate that whilst the pandemic disrupted urban lifestyles and increased urban‐to‐rural migration, shocks to the internal migration system do not seem to have led to an urban exodus.

We provided evidence revealing that the largest Spanish cities experienced the highest increase in migration outflows and declines in migration inflows, especially Madrid and Barcelona, as it has been also observed in Stockholm in Sweden (Vogiazides & Kawalerowicz, 2022), Tokyo in Japan (Fielding & Ishikawa, 2021), the chief cities of Germany (Stawarz et al., 2022) and densely populous areas in Britain (Rowe et al. 2022). Out‐migration away from these cities may have been linked to the fact that large cities became the main early epicentres of COVID‐19 infections and mortality (Florida et al., 2021; Rodríguez‐Pose & Burlina, 2021). High population density in cities has been linked to high spreading of COVID‐19 infections (Bhadra et al., 2021; Wong & Li, 2020). Out‐migration from cities has also been connected to high income households relocating to second and vacation residences elsewhere (Hughes, 2020; Paybarah et al., 2020).

Our findings indicated that out‐migration flows from cities to less populated areas headed towards rural destinations with a particular set of attributes. These destinations tended to comprise key touristic locations, including coastal towns, mountain areas and localities in close proximity to cities and natural parks. High concentration of second residences seem to have been a key factor underpinning the attractiveness of these locations (Alario et al., 2014; López‐Colás & Módenes, 2004).

Despite remarkable changes, we presented evidence suggesting that these alterations have been temporary. The scale of internal migration appeared to have returned to prepandemic levels over 2020. While internal migration flows and rates recorded pronounced changes during the early phases of the pandemic, as lockdowns were imposed and COVID‐19 rapidly spread across the country, they tended to converge to the levels observed during the four preceding years during the late months of 2020.

Taken together, we presented evidence of marked changes in the patterns of internal migrations during 2020. Yet, the COVID‐19 pandemic does not seem to have significantly altered existing structures in the internal migration system. Spanish cities have remained and are likely to remain key centres of attraction for migration flows. Agglomeration economies will probably continue to facilitate economic prosperity and thus promote the spatial concentration of population and businesses (Storper & Venables, 2004). At the same time, rural areas may lack the necessary infrastructure and services to support hybrid work arrangements. Telework is likely to become a more predominant form of interaction. Poor broadband connectivity, transport accessibility and the remoteness of rural locations are likely to represent major challenges to enable hybrid working (Chen & Wellman, 2004).

We analysed how the COVID‐19 pandemic led to changes in the spatial patterns of internal migration. Future research is needed to establish the profile of internal migrants and key socioeconomic factors underpinning these trends. The pandemic has had a very differentiated impact across the population and only privileged groups have reportedly been able to migrate. Particularly, these groups likely included wealthy individuals with second homes away from cities, and people in nonpublic facing and highly flexible jobs with telework capacity. As a consequence of the stringency measures and telework, second residence tenure and socioeconomic status seem to have been key factors underpinning changes in the direction of migration flows. Understanding and establishing the causal effect of these factors on internal migration is important to guide policies targeting to increase the attractiveness of rural locations as places of residence. Drawing on the 2021 Spanish Census and the EVR 2021 data when they become available, future research should also extend our analysis to examine the continuity or change in the patterns of internal migration flows, to establish the durability of the shifts over the COVID‐19 pandemic in 2020. While mass vaccination has reduced mortality and risk of infection, new variants have continued to cause anxiety, social discontent and rises in COVID‐19 cases. The net impact of these potentially conflicting effects on internal migration are to be established.

We used administrative data from the Spanish population register (the EVR) to capture internal migration movements. As indicated in Section 3, these records were impacted by under‐reporting of changes in residence during the early stages of the pandemic due to closure of registration offices, and they may have been affected by over‐reporting due to untruthful residential registrations to avoid selective local and regional mobility restrictions. While we believe that the data provide a relatively accurate representation of the internal migration trends during 2020, the spatial distribution of these reporting issues remained unknown and difficult to unpack. Future work could examine the spatial patterns of internal migration drawing on nontraditional data sources, such as mobile phone location data from applications (Rowe 2022; Rowe, Arribas‐Bel, et al., 2022), to substantiate the evidence provided here.

## CONFLICT OF INTEREST

The author declares no conflict of interest.

## Supporting information

Supplementary information.
