# Supplementary material for: Understanding patterns of internal migration during the COVID‐19 pandemic in Spain
Source: Popul Space Place. 2022 Jun 16;28(6):e2578. doi: 10.1002/psp.2578 (PMC9350359; doi:10.1002/psp.2578)
Supplement: Supplementary file 1 — Supplementary information. [file PSP-28-0-s001.pdf]

Supporting Information Material

Table 1. Number of municipalities and total population size in 2020 by territorial typology

Type of municipality	Number of municipalities	%	Population in 2020	%
Core cities	79	1.0 %	17,896,020	37.7 %
Suburbs	662	8.1 %	14,261,728	30.1 %
Town	352	4.3 %	7,299,486	15.4 %
Rural areas	7,038	86.6 %	7,993,561	16.8 %
**Total**	**8,131**	**100.0 %**	**47,450,795**	**100.0 %**

Source: Own elaboration using data from the INE.

Table 2. Out-, in- and internal net-migration rates in selected core city by origin and destination types: 2016-2019 (annual average) and 2020

City	Migration from or to:	Out-migration		In-migration		Net-migration	
		2016-2019	2020	2016-2019	2020	2016-2019	2020
Madrid	Rural areas	4.4	7.6	4.3	3.0	-0.2	-4.6
	Other urban areas	8.5	10.2	11.1	8.2	2.5	-1.9
	Suburbs	11.8	12.1	8.8	7.4	-3.1	-4.6
	Towns	2.5	3.2	2.7	2.0	0.2	-1.2
	**Total**	**27.4**	**33.1**	**26.8**	**20.7**	**-0.5**	**-12.4**
Barcelona	Rural areas	3.0	5.1	2.5	1.9	-0.5	-3.2
	Other urban areas	7.7	8.3	8.1	6.8	0.4	-1.5
	Suburbs	19.5	20.2	13.9	12.4	-5.6	-7.8
	Towns	2.4	3.2	2.3	1.7	-0.2	-1.5
	**Total**	**32.5**	**36.8**	**26.8**	**22.8**	**-5.8**	**-14.1**
Valencia	Rural areas	4.2	5.6	4.2	2.6	0.0	-3.0
	Other urban areas	9.2	7.4	9.8	8.3	0.7	0.8
	Suburbs	12.0	11.9	9.3	6.9	-2.8	-5.0
	Towns	5.0	5.2	4.8	3.4	-0.2	-1.8
	**Total**	**23.2**	**24.8**	**20.9**	**19.2**	**-2.3**	**-5.6**
Zaragoza	Rural areas	5.3	8.1	6.4	4.8	1.1	-3.3
	Other urban areas	8.3	7.3	8.3	7.0	0.0	-0.2
	Suburbs	3.1	3.7	2.8	2.4	-0.3	-1.4
	Towns	1.9	1.9	2.5	1.9	0.6	0.1
	**Total**	**18.6**	**21.0**	**20.0**	**16.1**	**1.4**	**-4.9**
Bilbao	Rural areas	4.6	6.6	4.9	3.9	0.2	-2.7
	Other urban areas	7.5	6.7	8.1	7.4	0.6	0.7
	Suburbs	13.6	14.9	12.6	11.1	-1.0	-3.8
	Towns	3.3	4.3	4.2	3.6	0.9	-0.7
	**Total**	**29.1**	**32.5**	**29.8**	**25.9**	**0.7**	**-6.5**

Source: Own elaboration using data from the Estadística de Variaciones Residenciales and Cifras Oficiales de Población (INE).

Figure 1. Internal out-, in- and net-migration rates in core cities with more than 250.000 inhabitants by origin and destination types: 2016-2019 (annual average) and 2020

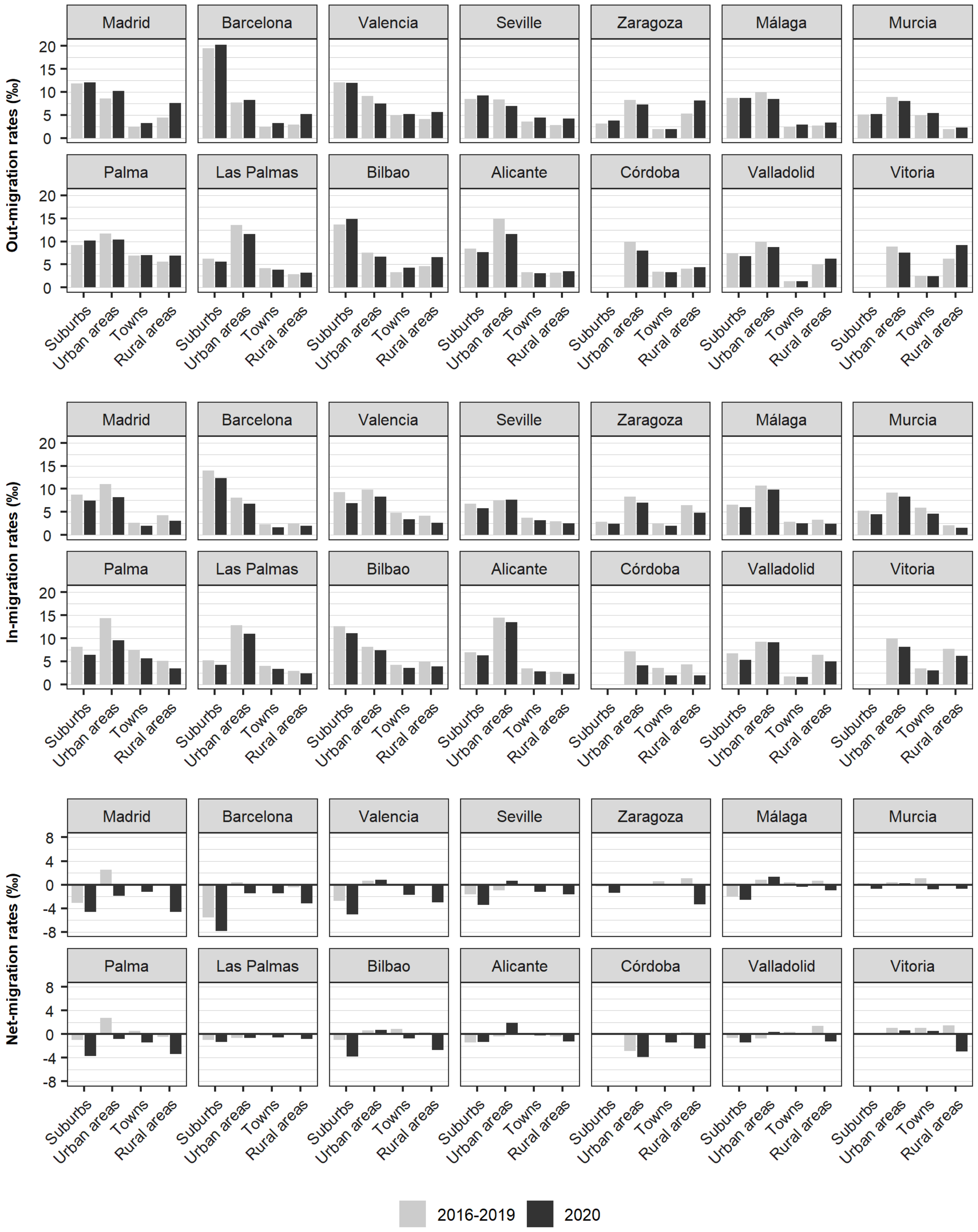

Source: Own elaboration using data from the Estadística de *Variaciones Residenciales* and *Cifras Oficiales de Población* (INE).

Figure 2. Internal out-migration rate from the selected core cities by type of municipality of destination and linear distance: 2016-2019 (annual average) and 2020

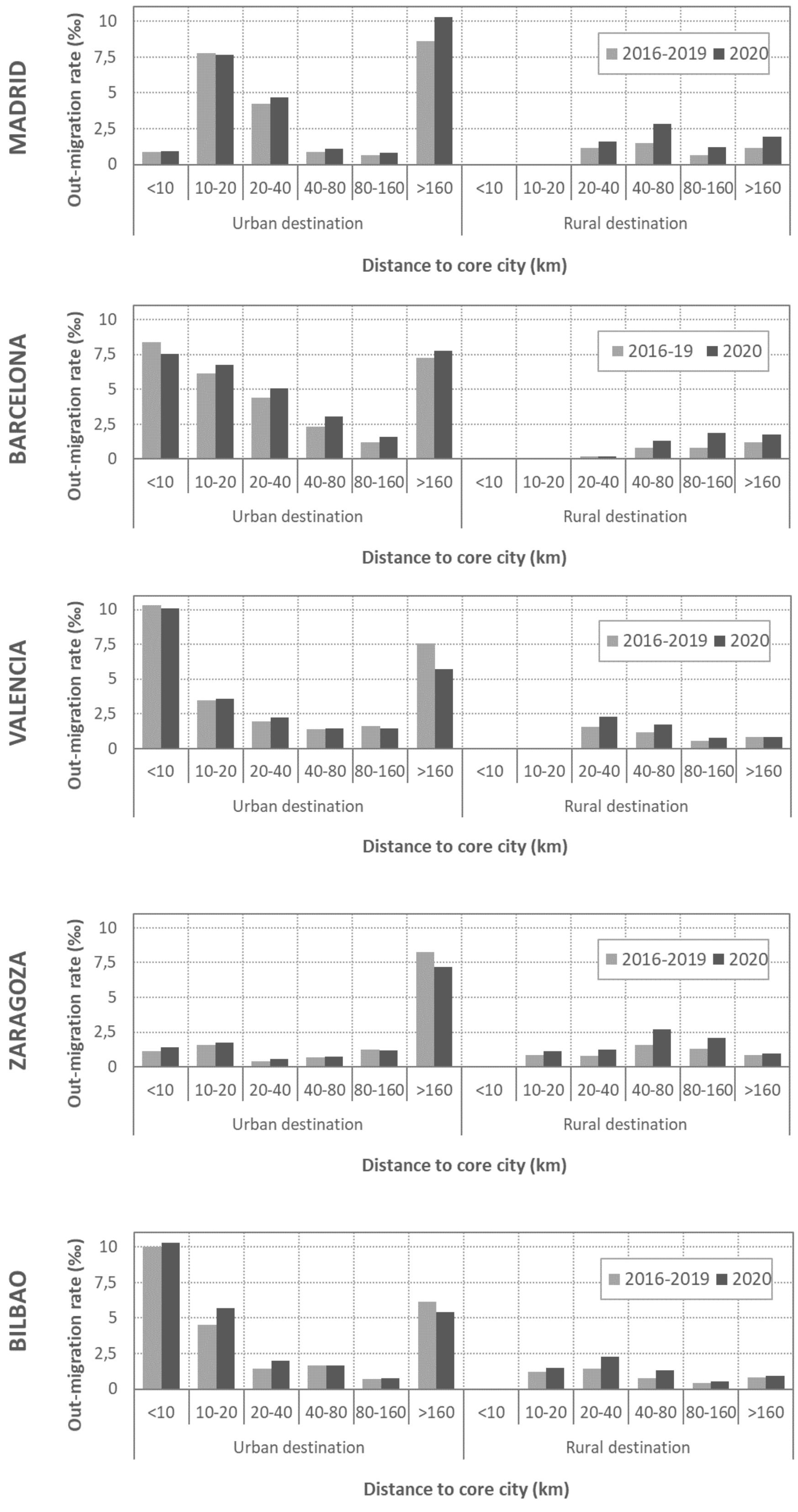

Source: Own elaboration using data from the Estadística de *Variaciones Residenciales* and *Cifras Oficiales de Población* (INE).

Figure 3. In-migration rates by municipality for out-migrants of selected cities (%): 2016-2019 (annual average) and 2020

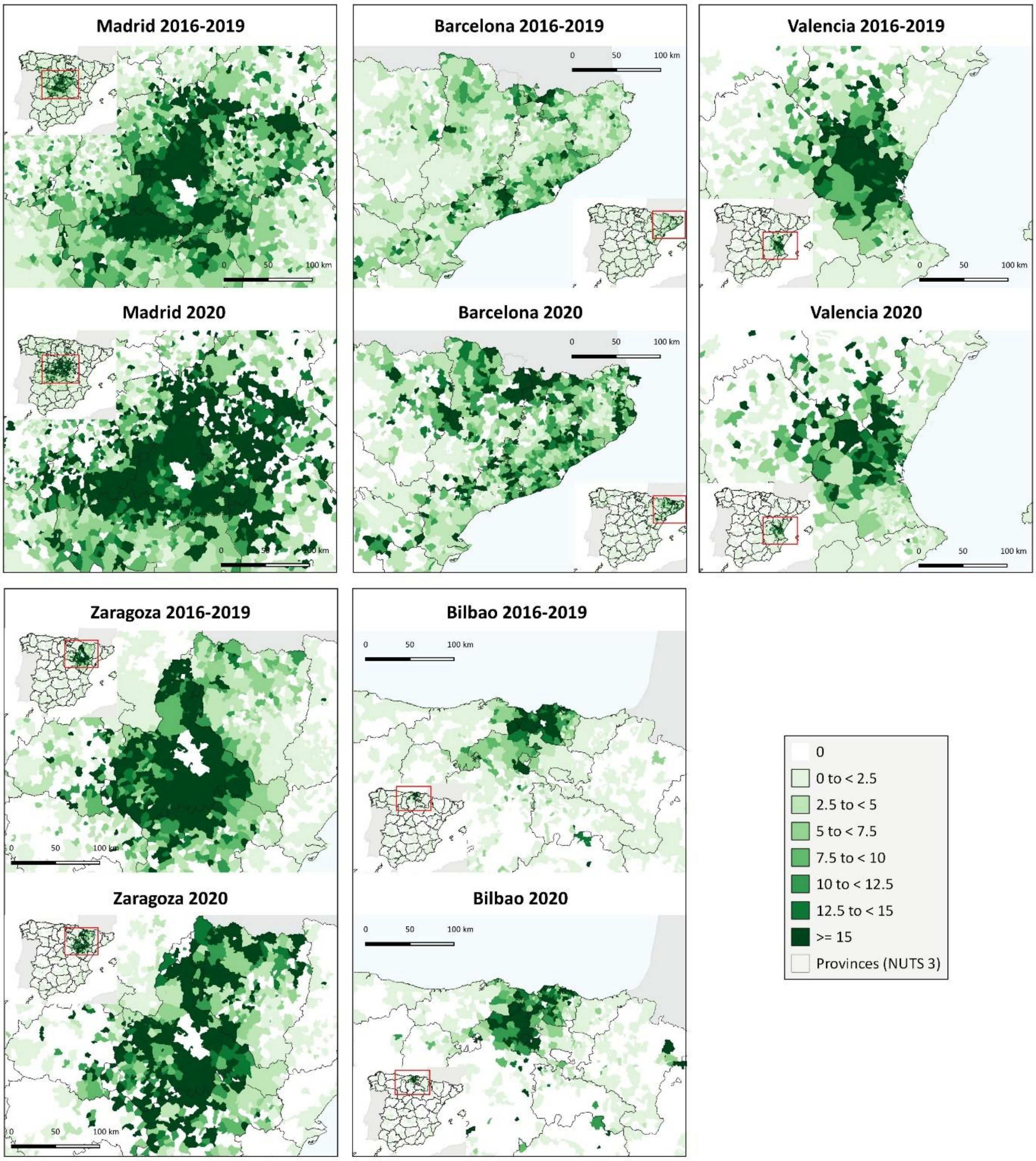

Source: Own elaboration using data from the Estadística de *Variaciones Residenciales* and *Cifras Oficiales de Población* (INE).
